# Methyl *N*-{4-[(4-meth­oxy­phen­oxy)meth­yl]-2-oxo-2*H*-chromen-7-yl}carbamate

**DOI:** 10.1107/S160053681202048X

**Published:** 2012-05-16

**Authors:** K. Mahesh Kumar, N. M. Mahabaleshwaraiah, O. Kotresh, S Jeyaseelan, H. C. Devarajegowda

**Affiliations:** a Department of Chemistry, Karnatak Science College, Dharwad 580 001, Karnataka, India; b Department of Physics, Yuvaraja’s College (Constituent College), University of Mysore, Mysore 570 005, Karnataka, India

## Abstract

In the title compound, C_19_H_17_NO_6_, the dihedral angle between the 2*H*-chromene ring system and benzene ring is 5.34 (6)°. A short intra­molecular C—H⋯O contact occurs. In the crystal, mol­ecules are linked by N—H⋯O hydrogen bonds, generating *C*(8) chains propagating in [010]. The chains are linked by C—H⋯O inter­actions and the packing also exhibits π–π stacking inter­actions between benzene and pyran rings, with a centroid–centroid distance of 3.676 (9) Å.

## Related literature

For a related structure and background to coumarins, see: Mahabaleshwaraiah *et al.* (2012 ▶). For further synthetic details, see: Kulkarni & Patil (1981 ▶).
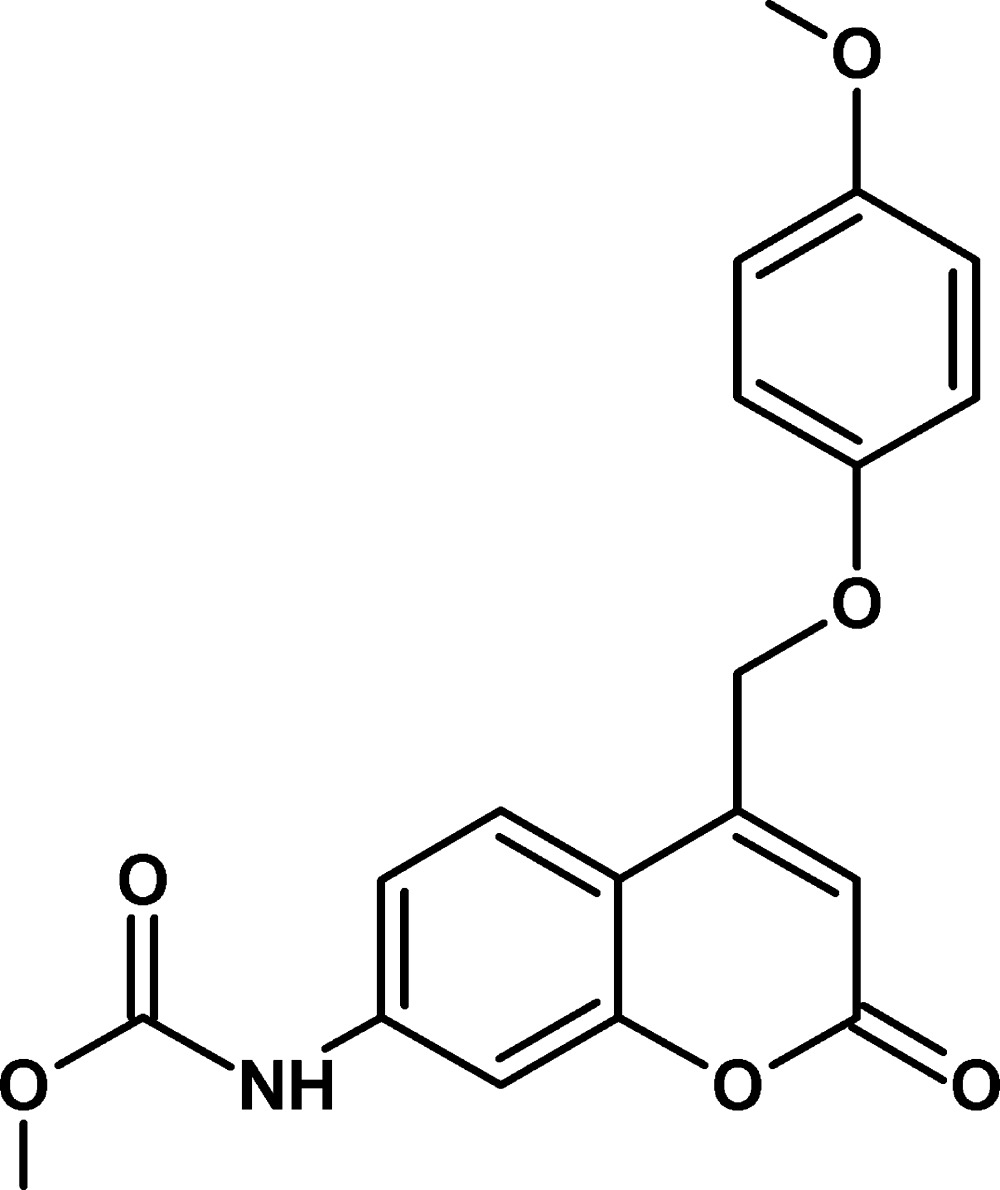


## Experimental

### 

#### Crystal data

C_19_H_17_NO_6_
*M* *_r_* = 355.34
Monoclinic, 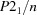

*a* = 8.3141 (1) Å
*b* = 17.3978 (3) Å
*c* = 11.5729 (2) Å
β = 94.309 (1)°
*V* = 1669.25 (5) Å^3^
*Z* = 4
Mo *K*α radiation
μ = 0.11 mm^−1^
*T* = 293 K
0.24 × 0.20 × 0.12 mm

#### Data collection

Bruker SMART CCD diffractometer
Absorption correction: multi-scan (*SADABS*; Sheldrick, 2007 ▶) *T*_min_ = 0.770, *T*_max_ = 1.000
14973 measured reflections
2939 independent reflections
2374 reflections with *I* > 2σ(*I*)
*R*_int_ = 0.023

#### Refinement

*R*[*F* ^2^ > 2σ(*F* ^2^)] = 0.035
*wR*(*F* ^2^) = 0.103
*S* = 1.06
2939 reflections
238 parameters
H-atom parameters constrained
Δρ_max_ = 0.14 e Å^−3^
Δρ_min_ = −0.12 e Å^−3^

### 

Data collection: *SMART* (Bruker, 2001 ▶); cell refinement: *SAINT* (Bruker, 2001 ▶); data reduction: *SAINT*; program(s) used to solve structure: *SHELXS97* (Sheldrick, 2008 ▶); program(s) used to refine structure: *SHELXL97* (Sheldrick, 2008 ▶); molecular graphics: *ORTEP-3* (Farrugia, 1997 ▶); software used to prepare material for publication: *SHELXL97*.

## Supplementary Material

Crystal structure: contains datablock(s) I, global. DOI: 10.1107/S160053681202048X/hb6754sup1.cif

Structure factors: contains datablock(s) I. DOI: 10.1107/S160053681202048X/hb6754Isup2.hkl

Supplementary material file. DOI: 10.1107/S160053681202048X/hb6754Isup3.cml

Additional supplementary materials:  crystallographic information; 3D view; checkCIF report

## Figures and Tables

**Table 1:** Hydrogen-bond geometry (Å, °)

*D*—H⋯*A*	*D*—H	H⋯*A*	*D*⋯*A*	*D*—H⋯*A*
N7—H7⋯O2^i^	0.86	1.99	2.8428 (16)	170
C15—H15⋯O5	0.93	2.30	2.8764 (19)	120
C19—H19*A*⋯O5^ii^	0.97	2.53	3.476 (2)	165

Symmetry codes: (i) 
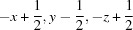
; (ii) 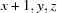.

**Fig. 1.**
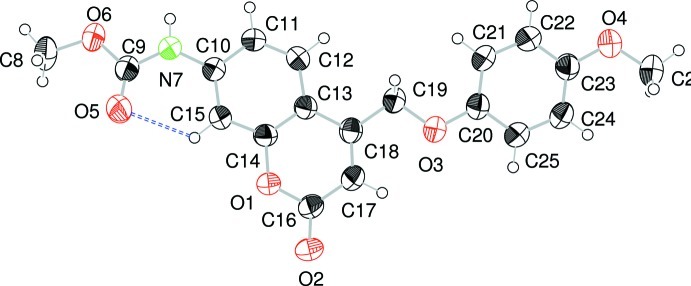
The molecular structure of the title compound. Displacement ellipsoids are drawn at the 50% probability level. Dashed lines indicate intramolecular hydrogen bonds.

**Fig. 2.**
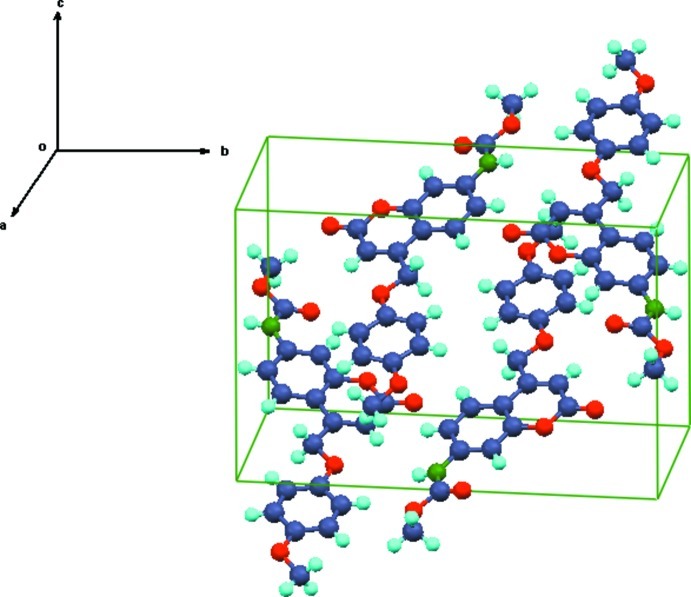
The packing of the molecules in the title structure.

## supplementary crystallographic information

### Comment

As part of our ongoing studies of coumarin derivatives with possible biological activity (Mahabaleshwaraiah *et al.*, 2012), we now describe the structure of the title compound (Fig. 1).

The 2*H*-chromene (O1/C10–C18) and benzene (C20–C25) rings are almost coplanar; the dihedral angle between them is 5.34 (6)°. In the crystal, (Fig. 2), the molecules are connected by C19—H19A···O5 and N7—H7···O2 interaction hydrogen bonds.(Table 1) Furthermore, the crystal structure features π-π stacking interactions between pyran*Cg2* and benzene*Cg3* rings, with a centroid *Cg2* (O3/C12–C16) -centroid *Cg3* (C13/C14/C17–C20) distance of 3.676 (9) Å.

### Experimental

The 4-bromomethyl coumarin required for the target molecule was synthesized according to an already reported (Kulkarni *et al.*1981) procedure involving Pechmann cyclization of phenols with 4-bromoethylacetoacetate. A mixture of 1.56 g (0.005 mol) of 7-carbonylamino-4-bromomethyl coumarin, 0.620 g (0.005 mol) of *p*-methoxy phenol and 0.70 g (0.005 mol) of powdered anhydrous K_2_CO_3_ in 30 ml of dry acetone were stirred at room temperature for 24 h. After completion of the reaction, the s eparated solid was filtered, washed with excess of dilute (10%) hydrochloric acid (50 ml) and then with an excess of cold water, dried and crystallized twice from ethanol & 1,4-dioxane mixture to yield colourless plates. Yield= 78%, *M*. P.475 K.

### Refinement

All H atoms were positioned at calculated positions N—H = 0.86 Å, C—H = 0.93 Å for aromatic H, C—H = 0.97 Å for methelene H and C—H = 0.96 Å for methyl H and refined using a riding model with *U*_iso_(H) = 1.5*U*_eq_(C)for methyl H and *U*_iso_(H) = 1.2*U*_eq_(C,*N*)for other H.

### Crystal data

**Table:**

C_19_H_17_NO_6_	*F*(000) = 744
*M**_r_* = 355.34	*D*_x_ = 1.414 Mg m^−^^3^
Monoclinic, *P*2_1_/*n*	Melting point: 475 K
Hall symbol: -P 2yn	Mo *K*α radiation, λ = 0.71073 Å
*a* = 8.3141 (1) Å	Cell parameters from 2939 reflections
*b* = 17.3978 (3) Å	θ = 2.1–25.0°
*c* = 11.5729 (2) Å	µ = 0.11 mm^−^^1^
β = 94.309 (1)°	*T* = 293 K
*V* = 1669.25 (5) Å^3^	Plate, colourless
*Z* = 4	0.24 × 0.20 × 0.12 mm

### Data collection

**Table:**

Bruker SMART CCD diffractometer	2939 independent reflections
Radiation source: fine-focus sealed tube	2374 reflections with *I* > 2σ(*I*)
Graphite monochromator	*R*_int_ = 0.023
ω and φ scans	θ_max_ = 25.0°, θ_min_ = 2.1°
Absorption correction: multi-scan (*SADABS*; Sheldrick, 2007)	*h* = −9→9
*T*_min_ = 0.770, *T*_max_ = 1.000	*k* = −20→20
14973 measured reflections	*l* = −13→13

### Refinement

**Table:**

Refinement on *F*^2^	Secondary atom site location: difference Fourier map
Least-squares matrix: full	Hydrogen site location: inferred from neighbouring sites
*R*[*F*^2^ > 2σ(*F*^2^)] = 0.035	H-atom parameters constrained
*wR*(*F*^2^) = 0.103	*w* = 1/[σ^2^(*F*_o_^2^) + (0.0518*P*)^2^ + 0.2287*P*] where *P* = (*F*_o_^2^ + 2*F*_c_^2^)/3
*S* = 1.06	(Δ/σ)_max_ < 0.001
2939 reflections	Δρ_max_ = 0.14 e Å^−^^3^
238 parameters	Δρ_min_ = −0.12 e Å^−^^3^
0 restraints	Extinction correction: *SHELXL97* (Sheldrick, 2008), Fc^*^=kFc[1+0.001xFc^2^λ^3^/sin(2θ)]^-1/4^
Primary atom site location: structure-invariant direct methods	Extinction coefficient: 0.0028 (9)

### Special details

**Table:**

Experimental. IR(KBr): 1067 cm^-1^(C—O—C), 1697 cm^-1^ (NH—C=O), 1727 cm^-1^ (Coumarin C=O), 3261 cm^-1^ (NH). GCMS: m/e: 355.1H NMR (500 MHz, DMSO.D6, \?, p.p.m.): 3.34 (s,3*H*, C_13_), 3.696(s,3*H*, C_1_), 5.2 (s,2*H*, C_6_), 6.38 (s 1H, C_17_), 6.88 (d,2*H*, C_3_ & C_15_), 7.04 (s,2*H*, C_4_ & C_14_), 7.38 (s,1*H*, C_19_), 7.56 (s,1*H*, C_10_),7.74 (s,1*H*, C_18_). Elemental analysis: C, 64.20; H, 4.78; N, 3.91; O, 27.19.
Geometry. All s.u.'s (except the s.u. in the dihedral angle between two l.s. planes) are estimated using the full covariance matrix. The cell s.u.'s are taken into account individually in the estimation of s.u.'s in distances, angles and torsion angles; correlations between s.u.'s in cell parameters are only used when they are defined by crystal symmetry. An approximate (isotropic) treatment of cell s.u.'s is used for estimating s.u.'s involving l.s. planes.
Refinement. Refinement of *F*^2^ against ALL reflections. The weighted *R*-factor *wR* and goodness of fit *S* are based on *F*^2^, conventional *R*-factors *R* are based on *F*, with *F* set to zero for negative *F*^2^. The threshold expression of *F*^2^ > 2σ(*F*^2^) is used only for calculating *R*-factors(gt) *etc*. and is not relevant to the choice of reflections for refinement. *R*-factors based on *F*^2^ are statistically about twice as large as those based on *F*, and *R*- factors based on ALL data will be even larger.

### Fractional atomic coordinates and isotropic or equivalent isotropic displacement parameters (Å^2^)

**Table:**

	*x*	*y*	*z*	*U*_iso_*/*U*_eq_	
O1	0.22869 (12)	0.25228 (5)	0.17803 (9)	0.0546 (3)	
O2	0.29518 (15)	0.36749 (6)	0.12426 (11)	0.0719 (4)	
O3	0.75136 (12)	0.21340 (6)	0.00648 (10)	0.0625 (3)	
O4	1.32758 (14)	0.12805 (7)	−0.16477 (12)	0.0738 (4)	
O5	−0.16647 (14)	0.08777 (7)	0.31601 (12)	0.0798 (4)	
O6	−0.15645 (12)	−0.02844 (7)	0.40139 (10)	0.0651 (3)	
N7	0.05554 (14)	0.01046 (7)	0.31564 (11)	0.0551 (3)	
H7	0.0887	−0.0347	0.3359	0.066*	
C8	−0.3183 (2)	−0.01795 (12)	0.43264 (17)	0.0758 (5)	
H8A	−0.3253	0.0291	0.4753	0.114*	
H8B	−0.3480	−0.0603	0.4798	0.114*	
H8C	−0.3901	−0.0157	0.3638	0.114*	
C9	−0.09510 (18)	0.02930 (9)	0.34198 (14)	0.0547 (4)	
C10	0.16316 (16)	0.05609 (8)	0.25940 (12)	0.0482 (3)	
C11	0.30076 (18)	0.02083 (9)	0.22107 (14)	0.0556 (4)	
H11	0.3157	−0.0318	0.2316	0.067*	
C12	0.41361 (18)	0.06269 (9)	0.16825 (14)	0.0548 (4)	
H12	0.5041	0.0380	0.1435	0.066*	
C13	0.39578 (16)	0.14200 (8)	0.15063 (12)	0.0465 (3)	
C14	0.25774 (17)	0.17482 (8)	0.18963 (12)	0.0468 (3)	
C15	0.14222 (17)	0.13397 (8)	0.24295 (13)	0.0503 (4)	
H15	0.0515	0.1586	0.2675	0.060*	
C16	0.33358 (18)	0.30026 (9)	0.12863 (14)	0.0544 (4)	
C17	0.47631 (18)	0.26706 (9)	0.08719 (14)	0.0534 (4)	
H17	0.5491	0.2987	0.0527	0.064*	
C18	0.50789 (16)	0.19144 (8)	0.09682 (12)	0.0486 (4)	
C19	0.65617 (17)	0.15584 (9)	0.05426 (14)	0.0543 (4)	
H19A	0.7178	0.1306	0.1179	0.065*	
H19B	0.6260	0.1175	−0.0042	0.065*	
C20	0.89376 (17)	0.18941 (9)	−0.03564 (13)	0.0515 (4)	
C21	0.94292 (19)	0.11408 (9)	−0.04124 (15)	0.0598 (4)	
H21	0.8782	0.0751	−0.0152	0.072*	
C22	1.08779 (19)	0.09621 (9)	−0.08528 (15)	0.0612 (4)	
H22	1.1200	0.0451	−0.0885	0.073*	
C23	1.18506 (18)	0.15266 (9)	−0.12443 (13)	0.0547 (4)	
C24	1.13658 (18)	0.22857 (9)	−0.11907 (13)	0.0563 (4)	
H24	1.2015	0.2674	−0.1453	0.068*	
C25	0.99133 (18)	0.24652 (9)	−0.07463 (13)	0.0552 (4)	
H25	0.9591	0.2976	−0.0710	0.066*	
C26	1.4187 (2)	0.18198 (11)	−0.22267 (18)	0.0783 (5)	
H26A	1.3534	0.2034	−0.2866	0.118*	
H26B	1.5110	0.1570	−0.2509	0.118*	
H26C	1.4538	0.2223	−0.1700	0.118*	

### Atomic displacement parameters (Å^2^)

**Table:**

	*U*^11^	*U*^22^	*U*^33^	*U*^12^	*U*^13^	*U*^23^
O1	0.0545 (6)	0.0394 (6)	0.0714 (7)	0.0001 (4)	0.0157 (5)	−0.0011 (5)
O2	0.0771 (8)	0.0378 (6)	0.1031 (10)	−0.0030 (5)	0.0219 (7)	−0.0017 (6)
O3	0.0518 (6)	0.0560 (6)	0.0820 (8)	−0.0025 (5)	0.0206 (6)	0.0081 (6)
O4	0.0598 (7)	0.0671 (8)	0.0982 (9)	0.0010 (6)	0.0296 (6)	0.0054 (6)
O5	0.0574 (7)	0.0748 (9)	0.1098 (10)	0.0136 (6)	0.0223 (7)	0.0293 (7)
O6	0.0536 (6)	0.0648 (7)	0.0789 (8)	−0.0073 (5)	0.0184 (5)	0.0110 (6)
N7	0.0505 (7)	0.0444 (7)	0.0720 (9)	0.0020 (5)	0.0150 (6)	0.0087 (6)
C8	0.0518 (10)	0.0929 (14)	0.0848 (13)	−0.0122 (9)	0.0191 (9)	0.0062 (10)
C9	0.0506 (9)	0.0549 (10)	0.0594 (9)	−0.0030 (7)	0.0082 (7)	0.0061 (7)
C10	0.0459 (8)	0.0465 (8)	0.0527 (8)	−0.0009 (6)	0.0060 (6)	0.0028 (6)
C11	0.0523 (8)	0.0436 (8)	0.0720 (10)	0.0045 (6)	0.0119 (7)	0.0063 (7)
C12	0.0474 (8)	0.0506 (9)	0.0677 (10)	0.0064 (6)	0.0129 (7)	0.0040 (7)
C13	0.0432 (7)	0.0459 (8)	0.0504 (8)	−0.0012 (6)	0.0038 (6)	−0.0007 (6)
C14	0.0480 (8)	0.0402 (8)	0.0522 (8)	0.0009 (6)	0.0038 (6)	−0.0015 (6)
C15	0.0456 (8)	0.0475 (9)	0.0591 (9)	0.0016 (6)	0.0122 (7)	−0.0011 (7)
C16	0.0574 (9)	0.0429 (9)	0.0633 (10)	−0.0069 (7)	0.0070 (7)	−0.0035 (7)
C17	0.0507 (8)	0.0481 (9)	0.0619 (9)	−0.0091 (7)	0.0088 (7)	0.0007 (7)
C18	0.0432 (8)	0.0526 (9)	0.0499 (8)	−0.0042 (6)	0.0016 (6)	−0.0008 (6)
C19	0.0473 (8)	0.0540 (9)	0.0627 (9)	−0.0038 (7)	0.0109 (7)	0.0067 (7)
C20	0.0457 (8)	0.0563 (9)	0.0529 (8)	−0.0042 (7)	0.0058 (6)	0.0023 (7)
C21	0.0552 (9)	0.0511 (9)	0.0746 (11)	−0.0092 (7)	0.0153 (8)	0.0063 (8)
C22	0.0600 (10)	0.0493 (9)	0.0753 (11)	−0.0024 (7)	0.0126 (8)	0.0024 (8)
C23	0.0493 (8)	0.0577 (10)	0.0576 (9)	−0.0024 (7)	0.0077 (7)	0.0005 (7)
C24	0.0536 (9)	0.0550 (9)	0.0612 (9)	−0.0106 (7)	0.0093 (7)	0.0059 (7)
C25	0.0557 (9)	0.0488 (9)	0.0615 (9)	−0.0045 (7)	0.0076 (7)	0.0037 (7)
C26	0.0662 (11)	0.0834 (13)	0.0892 (13)	−0.0052 (9)	0.0309 (10)	0.0102 (10)

### Geometric parameters (Å, º)

**Table:**

O1—C16	1.3636 (17)	C13—C18	1.4435 (19)
O1—C14	1.3738 (16)	C14—C15	1.3774 (19)
O2—C16	1.2124 (18)	C15—H15	0.9300
O3—C20	1.3786 (18)	C16—C17	1.434 (2)
O3—C19	1.4144 (17)	C17—C18	1.345 (2)
O4—C23	1.3742 (18)	C17—H17	0.9300
O4—C26	1.407 (2)	C18—C19	1.496 (2)
O5—C9	1.2042 (19)	C19—H19A	0.9700
O6—C9	1.3397 (18)	C19—H19B	0.9700
O6—C8	1.4309 (19)	C20—C21	1.376 (2)
N7—C9	1.3514 (19)	C20—C25	1.380 (2)
N7—C10	1.3935 (18)	C21—C22	1.378 (2)
N7—H7	0.8600	C21—H21	0.9300
C8—H8A	0.9600	C22—C23	1.371 (2)
C8—H8B	0.9600	C22—H22	0.9300
C8—H8C	0.9600	C23—C24	1.384 (2)
C10—C15	1.377 (2)	C24—C25	1.383 (2)
C10—C11	1.3993 (19)	C24—H24	0.9300
C11—C12	1.368 (2)	C25—H25	0.9300
C11—H11	0.9300	C26—H26A	0.9600
C12—C13	1.401 (2)	C26—H26B	0.9600
C12—H12	0.9300	C26—H26C	0.9600
C13—C14	1.3874 (19)		
			
C16—O1—C14	121.89 (11)	C18—C17—C16	121.85 (14)
C20—O3—C19	116.39 (12)	C18—C17—H17	119.1
C23—O4—C26	117.57 (13)	C16—C17—H17	119.1
C9—O6—C8	115.81 (13)	C17—C18—C13	119.37 (13)
C9—N7—C10	127.28 (13)	C17—C18—C19	122.58 (13)
C9—N7—H7	116.4	C13—C18—C19	118.05 (13)
C10—N7—H7	116.4	O3—C19—C18	109.56 (12)
O6—C8—H8A	109.5	O3—C19—H19A	109.8
O6—C8—H8B	109.5	C18—C19—H19A	109.8
H8A—C8—H8B	109.5	O3—C19—H19B	109.8
O6—C8—H8C	109.5	C18—C19—H19B	109.8
H8A—C8—H8C	109.5	H19A—C19—H19B	108.2
H8B—C8—H8C	109.5	C21—C20—O3	124.84 (13)
O5—C9—O6	124.20 (14)	C21—C20—C25	119.12 (14)
O5—C9—N7	126.60 (14)	O3—C20—C25	116.05 (14)
O6—C9—N7	109.20 (13)	C20—C21—C22	120.22 (15)
C15—C10—N7	123.09 (13)	C20—C21—H21	119.9
C15—C10—C11	119.03 (13)	C22—C21—H21	119.9
N7—C10—C11	117.86 (13)	C23—C22—C21	120.96 (15)
C12—C11—C10	120.86 (14)	C23—C22—H22	119.5
C12—C11—H11	119.6	C21—C22—H22	119.5
C10—C11—H11	119.6	C22—C23—O4	115.74 (14)
C11—C12—C13	121.39 (14)	C22—C23—C24	119.20 (14)
C11—C12—H12	119.3	O4—C23—C24	125.05 (14)
C13—C12—H12	119.3	C25—C24—C23	119.85 (14)
C14—C13—C12	116.04 (13)	C25—C24—H24	120.1
C14—C13—C18	118.16 (13)	C23—C24—H24	120.1
C12—C13—C18	125.80 (13)	C20—C25—C24	120.66 (15)
O1—C14—C15	115.25 (12)	C20—C25—H25	119.7
O1—C14—C13	121.04 (12)	C24—C25—H25	119.7
C15—C14—C13	123.71 (13)	O4—C26—H26A	109.5
C10—C15—C14	118.97 (13)	O4—C26—H26B	109.5
C10—C15—H15	120.5	H26A—C26—H26B	109.5
C14—C15—H15	120.5	O4—C26—H26C	109.5
O2—C16—O1	115.68 (14)	H26A—C26—H26C	109.5
O2—C16—C17	126.62 (14)	H26B—C26—H26C	109.5
O1—C16—C17	117.70 (13)		

### Hydrogen-bond geometry (Å, º)

**Table:**

*D*—H···*A*	*D*—H	H···*A*	*D*···*A*	*D*—H···*A*
N7—H7···O2^i^	0.86	1.99	2.8428 (16)	170
C15—H15···O5	0.93	2.30	2.8764 (19)	120
C19—H19*A*···O5^ii^	0.97	2.53	3.476 (2)	165

Symmetry codes: (i) −*x*+1/2, *y*−1/2, −*z*+1/2; (ii) *x*+1, *y*, *z*.
